# Biosafety and Blood Compatibility of Graphene Oxide Particles in In Vitro Experiments

**DOI:** 10.3390/ma18092128

**Published:** 2025-05-06

**Authors:** Yuriy Prylutskyy, Patrycja Bełdzińska, Natalia Derewońko, Tetiana Halenova, Nataliia Raksha, Marcin Zakrzewski, Grzegorz Gołuński, Svitlana Prylutska, Uwe Ritter, Olexii Savchuk, Jacek Piosik

**Affiliations:** 1 ESC “Institute of Biology and Medicine”, Taras Shevchenko National University of Kyiv, 01601 Kyiv, Ukraine; prylut@ukr.net (Y.P.); galenovatanya@gmail.com (T.H.); nkudina@ukr.net (N.R.); olexiy.savchuk@ukr.net (O.S.); 2 Laboratory of Biophysics, University of Gdańsk, 80-307 Gdańsk, Poland; patrycja.beldzinska@phdstud.ug.edu.pl (P.B.); marcin.zakrzewski@phdstud.ug.edu.pl (M.Z.); 3 Laboratory of Recombinant Vaccines, University of Gdańsk, 80-307 Gdańsk, Poland; natalia.w.derewonko@outlook.com; 4 Faculty of Plant Protection, Biotechnology and Ecology, National University of Life and Environmental Science of Ukraine, 03041 Kyiv, Ukraine; psvit_1977@ukr.net; 5 Institute of Chemistry and Biotechnology, Technical University of Ilmenau, 98693 Ilmenau, Germany; uwe.ritter@tu-ilmenau.de

**Keywords:** blood compatibility, dynamic light scattering, atomic force microscopy, rabbit platelet aggregation, rabbit plasma coagulation, Ames mutagenicity test, AlamarBlue cytotoxicity test, hemolytic assay, *Salmonella enterica* serovar Typhimurium

## Abstract

Graphene oxide (GO), owing to its extraordinary application prospects in biomedicine, is attracting growing research attention. However, the biosafety and blood compatibility of GO required for its clearance for use in clinical trials remain elusive. Therefore, we studied the mutagenic properties of GO as well as its cell toxicity and blood compatibility. Prior to biological experiments, we assessed the structural organization of GO using dynamic light scattering and microscopic visualization methods. The results of both the Ames mutagenicity test performed on *Salmonella enterica* serovar Typhimurium TA98 and TA102 strains and the cytotoxicity test on noncancerous, immortalized human keratinocytes revealed no mutagenic or toxic effects of GO. Simultaneously, GO reduced the viability of the MelJuSo human melanoma cell line. A blood compatibility assay revealed that a concentration of 10 μg/mL was critical for GO biosafety, as greater concentrations induced diverse side effects. Specifically, GO disrupts erythrocytes’ membranes in the dose-dependent manner. Moreover, GO at higher concentrations both inhibited the process of ADP (a physiological platelet agonist)-induced cell aggregation and affected their disaggregation process in platelet-rich plasma. However, in the blood clotting assessment, GO showed no effects on the activated partial thromboplastin time, prothrombin time, or thrombin time of the platelet-poor plasma. The obtained results clearly indicate that the relationship between the GO preparation method, its size, and concentration and biosafety must be cautiously monitored in the context of further possible biomedical applications.

## 1. Introduction

Graphene oxide (GO), is one of the novel carbon-based nanoparticles characterized as a graphene derivative. This nanomaterial is obtained by the processes of graphene oxidation and exfoliation [1]. One of the well-known properties of graphene is its layered structure. In the case of graphene oxide, each of the layers is formed by a lattice of sp^2^- and sp^3^-hybridized carbon atoms, with oxygen-based functional groups above and below the layer as well as around it, bound to the edges [2].

Interestingly, the GO synthesis method is the main factor behind the nanomaterial’s physicochemical properties. Namely, the synthesis approach affects GO’s oxidation state directly, which in turn is responsible for both the number and type of oxygen-based functional groups bound to the carbon scaffold of formed nanoparticles [3]. These unique properties of GO make it an excellent platform for extensive further modifications with other nanoparticles, metal ions, or even macromolecules [4]. Therefore, GO and its functionalized derivatives yield great potential for application in the fields of biotechnology, biomedicine and biomedical engineering, as well as other fields that include, but are not limited to, environmental protection, electronics, building, high-temperature materials, and membranes [3].

The prospective implementations of GO and its derivatives in biomedical fields include nanoplatforms for targeted drug delivery [5], biosensors [6], bioimaging sensors [7], and antiviral and antibacterial coatings [8]. Moreover, recent studies indicate that GO’s planar surface can serve as an astounding scaffold for production of stable nanocomposites with labeled antibodies, magnetic nanoparticles conjugated with anticancer agents, nucleic acids, peptides, or fluorescent probes [9,10]. Furthermore, several research groups have demonstrated the therapeutic potential of GO, which can not only modulate oxidative stress via anti-/pro-oxidant effects [11], but also promote tissue regeneration, enhancing cell differentiation [12,13].

Nevertheless, despite the progress observed in research on the application of GO, knowledge about the safety and effectiveness of GO still requires significant broadening. The fields that remain elusive embrace, among others, potential toxicity and blood compatibility of the GO—aspects that are always major concerns and risks in the consideration of nanomaterials for medical use.

The toxicity and health hazards of nanomaterials depend on the route of administration, nanomaterial composition and synthesis methods, surface modification, particle size, and shape [14,15]. The small size of nanoparticles facilitates their entry into the systemic circulation. Once there, they eventually contact various components of blood, either cells or proteins, and can potentially interfere with their physiological functions, leading to severe side effects. GO is composed of the same elements as human cells; however, its bi-dimensional nature may promote unique and distinctive interactions with virtually all biomacromolecules. These potential interactions, as yet unknown, may lead to significant alterations of homeostasis and, in consequence, immune system activation and hemotoxicity [16,17]. Moreover, Zhang et al. demonstrated that GO, in contrast to other carbon-based nanoparticles, is characterized by a long retention time and low uptake by the reticuloendothelial system [18].

High blood accumulation of GO and its potential to interact with blood components may lead to significant and prevalent systemic side effects. Therefore, the assessment of GO biocompatibility, mutagenicity and potential toxicity is required to facilitate further research on the potential use of GO and GO-based nanomaterials in the field of biomedicine. Hence, the current study concentrates on evaluation of biosafety and blood compatibility of GO in a series of in vitro experiments, starting with the Ames mutagenicity test via AlamarBlue cytotoxicity analysis and followed by hemolytic, platelet aggregation, and coagulation assays.

## 2. Materials and Methods

### 2.1. Chemicals

Sample of GO water dispersion (initial concentration 4 mg/mL) for the structural characterization and biological evaluation were kindly provided by Graphenea S.A. (San Sebastian, Spain).

Biological agar, nutrient agar, and nutrient broth media were acquired from BioMaxima S.A. (Gdansk, Poland). Histidine, biotin, ampicillin and tetracycline, doxorubicin (Certified Reference Material), and cisplatin (≥98% purity, HPLC) used in the Ames test were purchased from Sigma Aldrich Chemical Company (St. Louis, MO, USA).

All substrates for cell culture were purchased from Sigma Aldrich Chemical Company (St. Louis, MO, USA). AlamarBlue dye was purchased from Bio-Rad company (Hercules, CA, USA).

ADP and Triton X-100 used in rabbits’ plasma analyses were purchased from Sigma Aldrich Chemical Company (St. Louis, MO, USA).

Commercial kits for PT, TT and aPTT measurement were purchased from LLC «EASTERN-UKRAINIAN TRADING COMPANY» (Kharkiv, Ukraine).

### 2.2. Dynamic Light Scattering (DLS)

Hydrodynamic diameter measurements were conducted in polystyrene cuvettes at 25 °C using a Zetasizer Nano ZS (Malvern Panalytical, Malvern, UK) with a He-Ne laser (633 nm, 4 mW), at a 173° scattering angle. GO water dispersion (initial concentration 20 ng/L) was analyzed after 2 h sonication, 16 h stirring, and subsequent 0.5 h sonication prior to the conducted experiments. All measurements were conducted in distilled water. The DLS spectra were recorded and stored in digital form.

### 2.3. Atomic Force Microscopy (AFM)

The structural study of GO water dispersion (initial concentration 20 ng/L) was carried out by employing AFM with the “Solver Pro M” system (NT-MDT, Apeldoorn, The Netherlands). A drop of the analyzed water dispersion was transferred on the atomically smooth substrate (a freshly cleaved surface of mica; SPI supplies, V-1 grade) for layer deposition and the solvent succumbed to complete evaporation. The AFM measurements were carried out in the amplitude modulation tapping mode using ‘RTESPA–150’ type probes (Bruker, Billerica, MA, USA).

### 2.4. Scanning Electron Microscopy (SEM)

A drop of GO water dispersion (initial concentration 20 ng/L) succumbed to complete evaporation of the solvent. The morphology of the dried sample was examined by SEM using an FEI/Philips (XL30 ESEM, Hillsboro, OR, USA) equipped with an energy dispersive X-ray spectrometer (SEM/EDS).

### 2.5. Ames Mutagenicity Assay

*Salmonella enterica* serovar Typhimurium TA98 and *Salmonella enterica* serovar Typhimurium TA102 were purchased from Xenometrics AG (Allschwil, Switzerland).

The Ames mutagenicity assay was performed with *Salmonella enterica* serovar Typhimurium TA98 and TA102 according to the procedure described previously [19,20], with modifications. Namely, a mixture containing 100 μL of the overnight bacteria culture, 50 μL of 3% (*v*/*v*) NaCl, and 100 μL of GO particles (2 h sonication (on ice, 200 W, 40 kHz), 16 h stirring, and, subsequently, 0.5 h sonication (on ice, 200 W, 40 kHz) prior to the conducted experiments; sterile distilled water as a negative control) was incubated for 4 h at 37 °C with shaking (GO final concentrations: 0.01, 0.04, 0.2, 1, 4, 20 ng/plate). Afterwards, the mixture was centrifuged for 5 min at 11,840× *g*, the pellet was washed with 0.75% NaCl, and it was resuspended in 300 μL of 0.75% (*v*/*v*) NaCl solution containing 0.1 μM histidine and 0.1 μM biotin. The bacterial suspension was spread on a glucose minimal agar plate and incubated in darkness for 48 h at 37 °C. All experiments were performed in triplicate. Doxorubicin (100 ng/plate) and cisplatin (200 ng/plate) were used as positive controls for *Salmonella enterica* serovars Typhimurium TA98 and TA102, respectively.

### 2.6. In Vitro Toxicity Assay

#### 2.6.1. Cell Culture

The HaCaT human immortalized keratinocytes cell line was cultivated in Dulbecco’s Modified Eagle’s Medium (DMEM) containing 4500 mg/L glucose supplemented with 10% (*v*/*v*) fetal bovine serum, 4 mM L-glutamine, 100 units/mL penicillin, 100 mg/mL streptomycin, and 0.25 µg/mL amphotericin B. The MelJuSo human melanoma cell line was cultured in Roswell Park Memorial Institute (RPMI) 1640 Medium supplemented with 10% (*v*/*v*) fetal bovine serum, 4 mM L-glutamine, 100 units/mL penicillin, 100 mg/mL streptomycin, and 0.25 µg/mL amphotericin B. All cell lines were maintained in a humidified atmosphere containing 5% CO_2_ at 37 °C.

#### 2.6.2. Cytotoxicity Assay

HaCaT and MelJuSo cells were seeded in a 96-well plate (2 × 10^4^ cells/well) and incubated in a humidified atmosphere containing 5% CO_2_ at 37 °C overnight. The cell cultures were then washed three times with media devoid of phosphate-buffered saline (PBS). Subsequently, diverse concentrations of GO (0.05–5 µg/well) in media devoid of PBS, after 2 h of sonication and 16 h of stirring, were added to the cell cultures and incubated for 24 h. The experiment was performed with three biological replicates to facilitate the significance of data. Untreated cells served as the negative control. Subsequently, 10 µL of AlamarBlue was added to each well 4 h prior to the end of the 24 h incubation. Absorbance was measured at the 570 nm and 600 nm wavelengths. Pure media were used as negative controls. The percentage of AlamarBlue reduction was calculated as the difference between treated and untreated cells, according to the manufacturer’s protocol:

$$diff\left(\%\right)=\frac{\left(O_{2}\times A_{1}\right)-(O_{1}\times A_{2})}{\left(O_{2}\times P_{1}\right)-(O_{1}\times P_{2})}$$

where diff—difference between treated and control cells; O_1_—ε_570_ of oxidized AlamarBlue (80586 M^−1^cm^−1^); O_2_—ε_600_ of oxidized AlamarBlue (117216 M^−1^cm^−1^); A_1_—absorbance of the test wells at 570 nm; A_2_—absorbance of test wells at 600 nm; P_1_—absorbance of control wells at 570 nm; P_2_—absorbance of control wells at 600 nm. Data were presented as the mean ± standard deviation.

### 2.7. Blood Compatibility Assessment

#### 2.7.1. Sample Preparation

Rabbits (2.50–3.50 kg) were purchased from the vivarium of the Educational and Scientific Center “Institute of Biology and Medicine”, Taras Shevchenko National University of Kyiv (Kyiv, Ukraine). The Bioethical Committee of the Taras Shevchenko National University of Kyiv approved animal care and procedures (protocol No. 9 dated 4 September 2023). Animal use was in accordance with the European Convention for the Protection of Vertebrate Animals Used for Experimental and Other Scientific purposes” (Strasbourg, 1986) and Article 26 of the Law of Ukraine “On the Protection of Animals from Cruelty” (No. 3447-IV, 21 February 2006), as well as the European Union Directive of 22 September 2010 (2010/63/EU) for the protection of animals used for scientific purposes.

Blood was collected from the rabbit’s ear artery into polyethylene tubes with 3.8% (*w*/*v*) sodium citrate to the ratio of 9:1. Platelet-rich plasma (PRP) for platelet analysis was obtained by centrifugation of stabilized blood at 150× *g* for 10 min at room temperature. Platelet-poor plasma (PPP) was prepared by further centrifugation of the remaining blood at 2500× *g* for 20 min at room temperature.

To evaluate the hemolytic activity of GO, a rabbit erythrocyte suspension was prepared. Briefly, rabbit blood stabilized with sodium citrate was centrifuged at 600× *g* for 10 min at 4 °C. Plasma and the white buffy coat were removed carefully, and erythrocytes were collected. Subsequently, the erythrocytes suspension was washed three times in PBS at a pH of 7.2. After final aspiration, the erythrocyte pellet was diluted 1:50 in PBS at a pH of 7.0 to obtain a 2% erythrocyte suspension. All the pH values were controlled and, if necessary, adjusted using a laboratory pH meter ADWA AD1020 (Adwa Instruments, Szeged, Hungary).

#### 2.7.2. Hemolytic Assay

The hemolytic activity of GO was tested in experiments in vitro according to the procedure described previously [21]. Specifically, 100 μL of GO solution (final concentrations: 0.01, 0.1, 1, 10, 50 and 100 µg/mL), distilled water (negative control), or 10% (*v*/*v*) Triton X-100 (positive control) were mixed with 900 μL of 2% erythrocyte suspension in an Eppendorf tube and incubated for 1 h at 37 °C. Subsequently, the tubes were centrifuged at 2000× *g* for 5 min, and 200 μL of the supernatant from each tube was transferred to a flat-bottom 96-well plate. Finally, the optical density (OD) of every sample was measured using a microplate spectrophotometer (BioTek Instruments Inc., Winooski, VT, USA) at the 541 nm wavelength. All experiments were performed in triplicate using blood samples from three rabbits. Finally, the values registered for samples treated with GO (OD*_GO_*) were normalized relative to positive (100% lysis; OD*_pos_*) and negative (OD*_neg_*) control samples to give the hemolysis ratio (HR) by using the following equation:

$$HR\;\left(\%\right)=\frac{\left(\;{OD}_{GO}-{OD}_{neg}\;\right)}{\left(\;{OD}_{pos}-{OD}_{neg}\;\right)}\times 100\%$$

#### 2.7.3. Platelet Aggregation Assay

The effect of GO on in vitro aggregation of rabbit platelets was tested by means of a photo-optical aggregometer AT-02 (Medtech, Minsk, Belarus). The platelet count in PRP was adjusted to about 2.3–2.5 × 10^5^ cells/μL before the procedure using pure plasma. Subsequently, PRP was pre-incubated with GO (final concentrations: 1, 5, 10, 50, and 100 µg/mL of PRP) at 37 °C with continuous stirring at 600 rpm. Analyzed mixtures were sampled after 5 min and 1 h of incubation. Afterwards, aggregation of platelets was induced by adding ADP (final concentration 5 µM)—an agonist promoting platelet activation—and platelet aggregation was recorded for at least 7 min. Obtained aggregation curves were analyzed and four parameters were estimated: (1) maximum degree of aggregation (*A_max_*) was determined by measuring the maximum height of the aggregation wave over a 4 min period beginning at the onset of platelet aggregation; (2) initial velocity of aggregation (*V*_0_) was determined by drawing a line tangent through the steepest linear part of the aggregation tracing and determining the slope from 1 point along the curve (the slope of this tangent was expressed in %·s^−1^); (3) time needed to reach maximal aggregation (*T_max_*) in seconds; (4) aggregation level at 6 min after the agonist supplementation (*A_6min_*), enabling estimation of the platelet disaggregation process. The curves representing the ADP-dependent aggregation after 5 min or 1 h of PRP incubation with distilled water (instead of GO) were used to calculate control parameters. Each measurement was performed in triplicate for plasma obtained from three individual rabbits.

#### 2.7.4. Coagulation Assay

To determine the GO influence on the blood coagulation prothrombin time (PT), thrombin time (TT), and activated partial thromboplastin time (aPTT) were evaluated by using a coagulation analyzer RT-2201C (Rayto, Shenzhen, China) and the commercial kits for PT, TT and aPTT measurement. Prior to the coagulation analysis, rabbit pure plasma was pre-incubated with different concentrations of GO (final concentrations: 0.01, 0.05, 0.1, 0.5, 1, 5 and 10 µg/mL of PPP) at 37 °C. Analyzed mixtures were sampled after 5 min and 1 h of incubation.

Subsequently, to check the clot formation in aPTT test, 50 μL of the tested sample was mixed with 50 μL of aPTT reagent in the coagulometric cuvette and incubated for 3 min at 37 °C. Clotting time in seconds was recorded after the addition of 50 μL of 0.025 M CaCl_2_. For TT assay 50 μL of the tested sample was mixed with 100 μL of thrombin (final activity 3 U/mL) in the coagulometric cuvette and time necessary for the clotting plug to form was recorded. The time required for the plasma to clot was measured in the PT test by mixing 50 μL of the tested sample with 100 μL of thromboplastin–calcium mixture. All coagulation tests were performed in triplicate using plasma from three rabbits. Plasma incubated for 5 min or 1 h with distilled water (instead of GO) was used as a control.

### 2.8. Statistical Analysis

Statistical analysis was performed with Statistica 14.0 (TIBCO Software Inc., Palo Alto, CA, USA) software. For the Ames test results, one-way analysis of variance (ANOVA) followed by the post-hoc RIR Tukey’s test was applied. AlamarBlue cytotoxicity test results were verified using a Mann–Whitney U test. The results of hemolytic assays were analyzed by employing an unpaired *t*-test. In every analysis, the significance level (*α*) was established at 0.05.

## 3. Results

### 3.1. Particle Characterization

**Figure 1:**
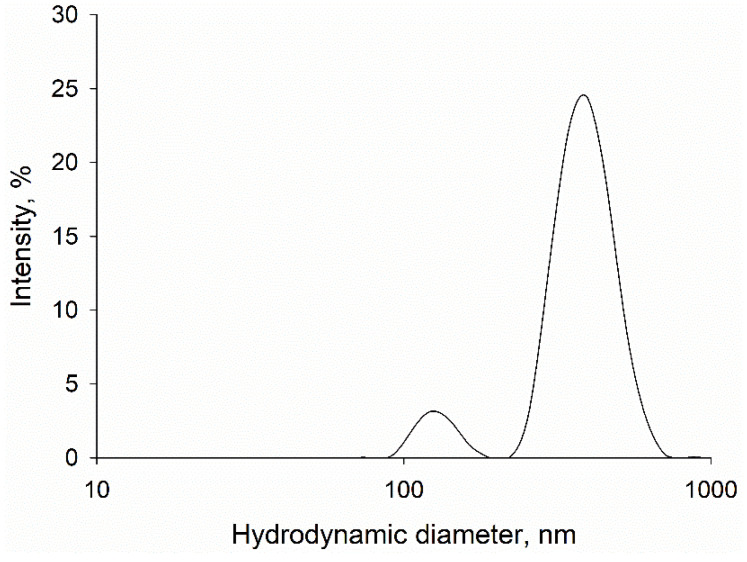
DLS analysis of GO water suspension (20 ng/L): hydrodynamic diameters of peaks: 128 ± 5 nm and 389 ± 25 nm; PdI: 0.29 ± 0.1.

We employed dynamic light scattering (DLS) to determine the average hydrodynamic diameter of GO particles. The obtained results revealed two main clusters of nanostructures with hydrodynamic diameters of 128 ± 5 nm and 389 ± 25 nm (Figure 1). We also established the polydispersity index (PdI) of the sample at the level of 0.29 ± 0.1, which indicates a low to moderate heterogeneity of the GO particles in the water dispersion.

**Figure 2:**
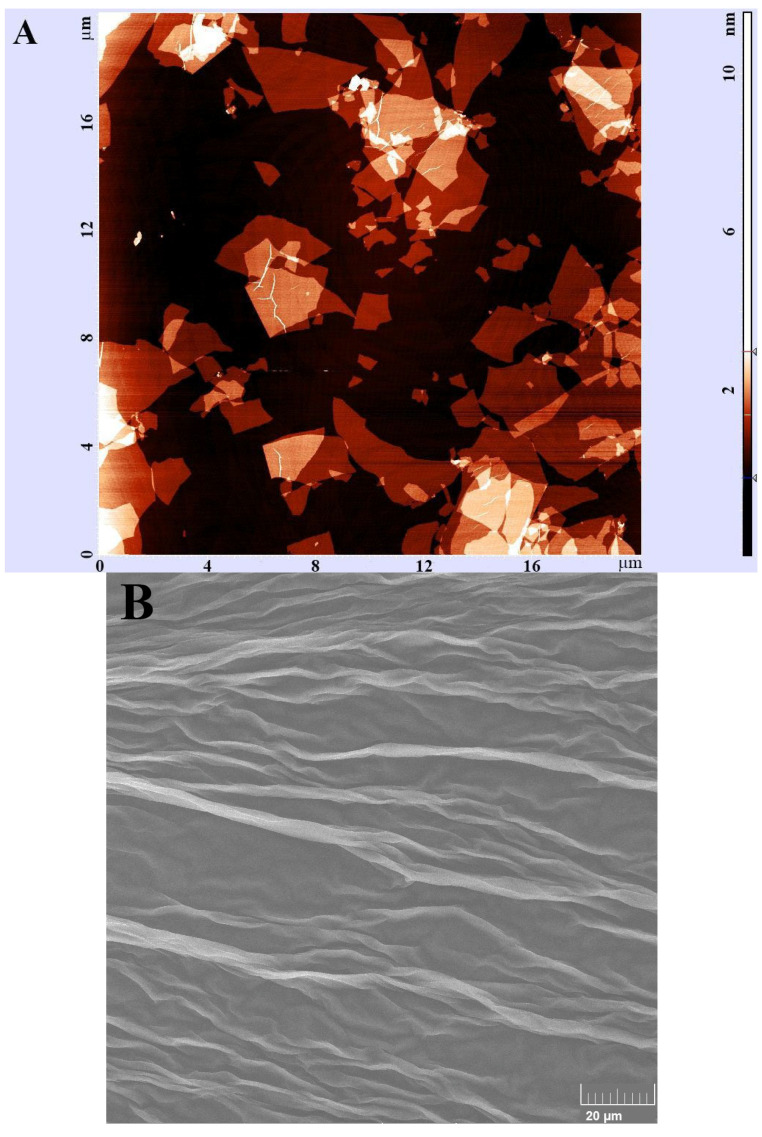
Microscopic imaging of graphene oxide. (**A**): AFM image of GO particles deposited from aqueous solution (20 ng/L) on the mica surface. (**B**): SEM image of GO monolayer (20 ng/L) on the Si(100) substrate.

We also visualized GO structures using atomic force microscopy (AFM) and scanning electron microscopy (SEM). The typical AFM depiction is presented in the Figure 2A: the lateral size of the nanoclusters does not exceed 4 µm, a result that is further confirmed by the SEM imaging of the GO monolayer (Figure 2B). However, particles thickness assessment based on the AFM height data may be affected by significant inaccuracies as a result of both possible solvent deposition between the mica substrate and GO layers and diverse interactions between the AFM probe and either the mica substrate or GO layers [22]. Therefore, to assess the height of the GO layer, we evaluated overlapping GO structures, measuring the height of the upper GO layer and relating it to the height of basal GO layer following the analysis method described before by Nemes-Incze et al. [23]. The particles’ height was established at 0.75–0.95 nm using this method. This value, when related to the interlayer gap of bulk graphite (approximately 0.355 nm), indicates bi- or trilayer GO particles deposited on the mica surface.

### 3.2. Ames Mutagenicity Test

**Figure 3:**
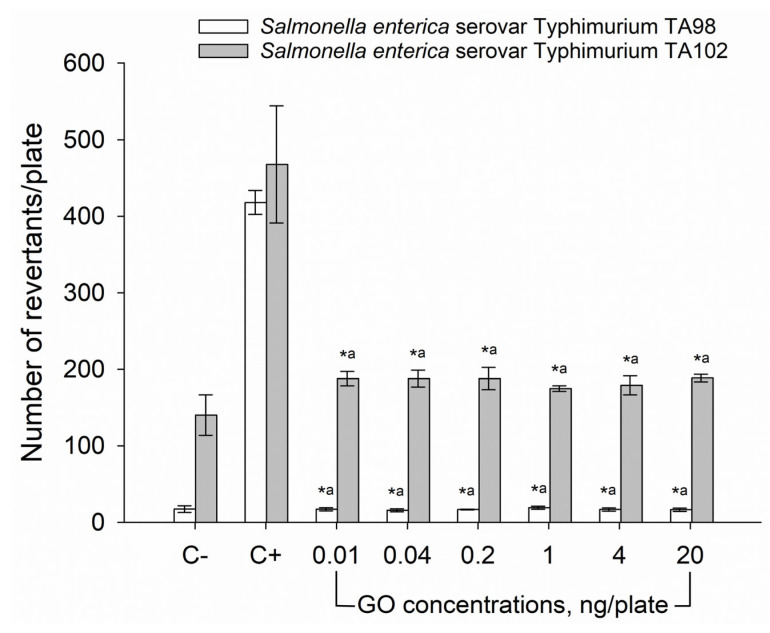
Mutagenic activity of GO particles in *Salmonella enterica* serovar Typhimurium Ames assay. TA98 strain—white columns; TA102 strain—gray columns. C− negative control (sterile water), C+ positive control (100 ng/plate doxorubicin for TA98 strain, 200 ng/plate cisplatin for TA102 strain). Results are reported as the average number of revertants ± standard deviation. * significant difference from the positive control for each bacterial strain separately (*p* < 0.05); ^a^ no significant difference from the negative control for each bacterial strain separately (*p* > 0.05).

Subsequently, we performed the Ames mutagenicity test using two bacterial strains—*Salmonella enterica* serovar Typhimurium TA98 and *Salmonella enterica* serovar Typhimurium TA102—to assess whether GO particles have mutagenic properties. The obtained results, presented in Figure 3, show no significant differences in the number of revertants between the GO samples in any concentration (0.01–20 ng/plate) and negative controls, indicating a lack of GO mutagenicity.

### 3.3. AlamarBlue In Vitro Cytotoxicity Test

**Figure 4:**
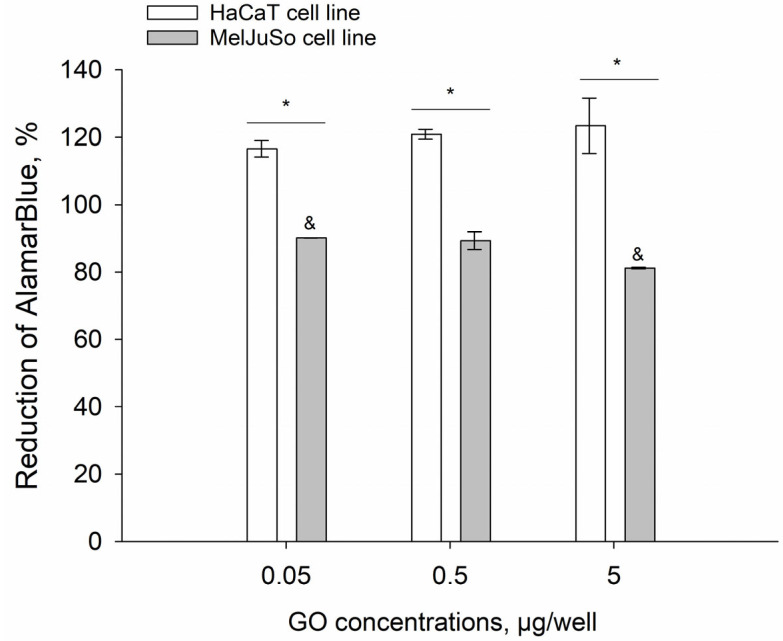
GO particles’ cytotoxic activity in the tested eukaryotic cell lines. Comparison of the cell viability modulation in HaCaT (white) and MelJuSo (gray) keratinocyte and melanoma cell lines. GO particles’ concentration range from 0.05–5 µg/well. Results are reported as the mean percentage difference between treated and untreated control ± standard deviation. * significant difference between both tested cell lines (*p* < 0.05) ^&^ significant difference between GO concentrations in MelJuSo cell line (*p* < 0.05).

Subsequently, we used two eukaryotic cell lines, HaCaT—a non-cancerous, human keratinocyte line and MelJuSo—a cancerous, human melanoma cell line, to evaluate the cytotoxicity of GO water dispersion. The obtained results reveal significant differences between GO effects in each of the analyzed lines. In the case of the MelJuSo cell line, we observed dose-dependent cytotoxicity of GO in the entire concentration range (viability reduced to 81–90%), while in the case of the HaCaT cell line, a small increase in cell viability was recorded (Figure 4).

### 3.4. Blood Compatibility

#### 3.4.1. Hemolytic Activity

**Figure 5:**
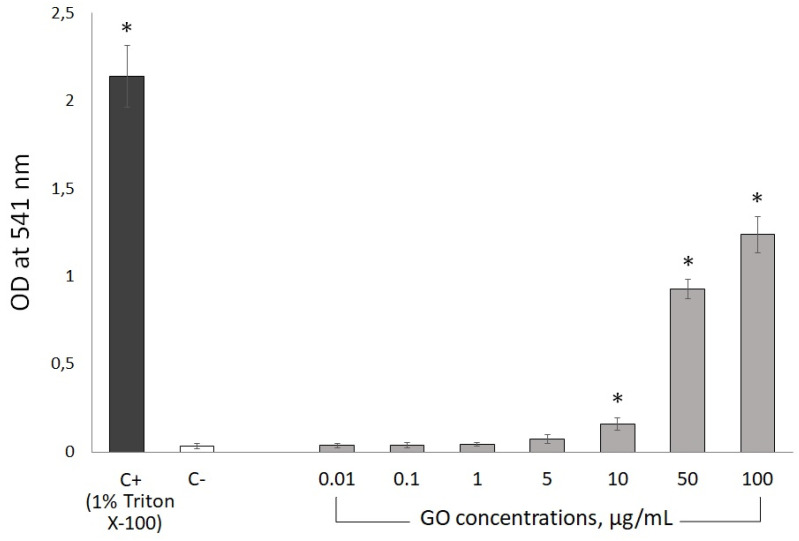
Hemolytic potential of GO, presented as OD_541_ (Y-axis) of free hemoglobin in a 2% rabbit erythrocyte solution incubated for 60 min at +37 °C with ultra-pure water (negative control, C−), 1% Triton X-100 (positive control, C+), or GO (at concentrations of 0.01, 0.1, 1, 5, 10, 50 and 100 µg/mL of cell suspension). Results are reported as the average values from three experimental replicates ± standard deviation. * significant difference from the negative control (*p* < 0.05).

Subsequent to the mutagenicity and toxicity experiments, we employed a series of assays to evaluate the blood compatibility of GO. Firstly, the potential of GO to induce hemolysis was analyzed to determine red blood cell (RBC) toxicity. The release of hemoglobin from the RBC incubated with GO was measured spectroscopically (at the wavelength 541 nm) and compared to the data recorded for cells incubated with detergent (1% Triton X-100). The obtained results indicate that GO at lower doses (0.01–5 µg/mL) does not disrupt RBC membranes significantly (Figure 5). Nevertheless, higher doses of GO (10–100 µg/mL) damage RBC membranes, inducing significant release of hemoglobin. The OD_541_ for the two highest GO concentrations, namely 50 µg/mL and 100 µg/mL, was measured at 42% and 57% of the positive control. The level of hemolysis induced by 10 µg/mL was relatively lower (6% of positive control); however, it was still significantly different from the negative control (Figure 5).

#### 3.4.2. Platelet-Rich Plasma (PRP) Aggregation Kinetics

**Figure 6:**
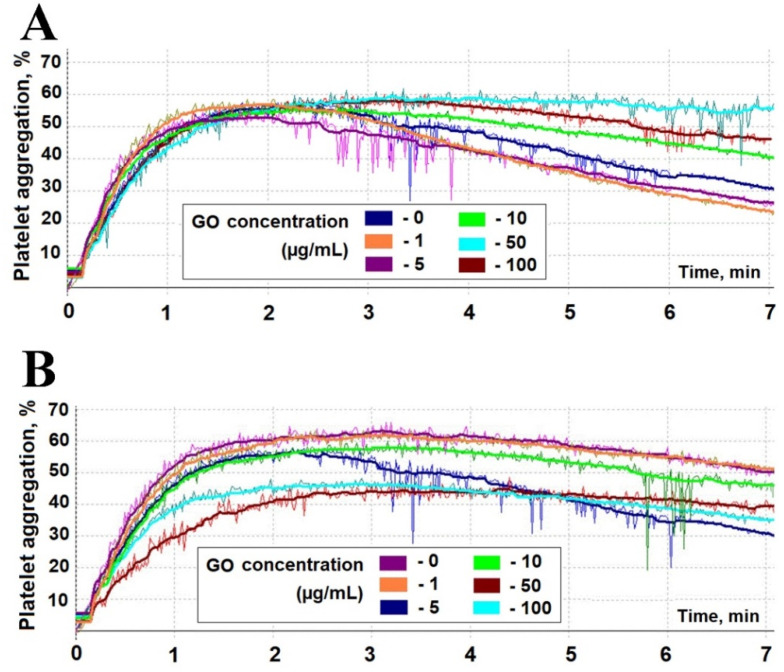
Platelet aggregation curves obtained in rabbit PRP after 5 min (**A**) and 1 h (**B**) incubation with GO (concentrations: 0, 1, 5, 10, 50, and 100 µg/mL of PRP) after induction with 5 µM ADP.

Subsequently, we investigated platelet aggregation, another important parameter in assessing biocompatibility of relatively large structures, such as GO nanoparticles. To assess GO’s influence on ADP-induced PRP aggregation kinetics, we analyzed rabbit PRP incubated with five GO concentrations (1, 5, 10, 50 and 100 µg/mL), sampled at 5 min and 1 h. The platelet aggregation curves after ADP induction are presented at Figure 6.

Subsequently, we calculated four parameters of platelet aggregation in order to further quantify GO’s influence on the process. We measured the maximum aggregation (*A_max_*), initial velocity of the process (*V*_0_), time required to reach maximal aggregation velocity (*T_max_*), and the aggregation level 6 min after the ADP supplementation (*A_6min_*). The values of the determined parameters (Table 1) indicate no influence of GO in the entire concentration range on all of the *A_max_*, *V*_0_, and *T_max_* in the samples taken after 5 min of PRP incubation with the analyzed structures. However, the values of *A_6min_* for the samples incubated for 5 min with 10, 50, and 100 µg/mL of GO are significantly elevated, revealing significant inhibition of the platelet disaggregation process by higher concentrations of GO.

Analysis of the samples taken after 1 h incubation of PRP with GO reveals a more prominent influence of the two highest concentrations of structures (50 and 100 µg/mL). However, the pattern was partially opposite from the data registered for samples taken after 5 min of incubation. In the case of 5 min incubation, *A_max_*, *V*_0_, and *T_max_* were unaffected, while in the case of 60 min, all three parameters were significantly changed. Specifically, *A_max_* values were reduced from 55% for the control to 45% and 43% for samples incubated with 50 µg/mL and 100 µ/mL of GO, respectively. Similarly, the *V*_0_ value was reduced from 0.9 to 0.6%·s^−1^; however, in this case, the highest GO concentration exhibited significant influence. Correspondingly, the values of *T_max_* were increased significantly, from 95 s to 115 s and 140 s for samples incubated with 50 µg/mL and 100 µ/mL of GO, respectively. Interestingly, in contrast to samples incubated for 5 min, *A_6min_* value was unaffected in the case of the highest GO concentrations, while 1 to 10 µg/mL of GO inhibited platelet disaggregation. Nevertheless, the results of PRP aggregation analysis indicate that the process may be affected by GO; however, it is mainly limited to the highest GO concentrations analyzed (50 and 100 µg/mL).

#### 3.4.3. Procoagulant Activity

Finally, we analyzed whether GO in a broad concentrations range (0.01, 0.05, 0.1, 0.5, 1, 5 and 10 µg/mL) induces the clot formation potential of platelet-poor plasma (PPP). We evaluated prothrombin time (PT), activated partial thromboplastin time (aPTT), and thrombin time (TT). The values of these parameters, presented in Table 2, indicate no influence of GO on clot formation, either after 5 min or 30 min incubation.

## 4. Discussion

Evaluating the properties of GO in a water dispersion is crucial in the context of its medicinal application. One of its crucial properties is the particle size, as this is reported to contribute to and affect the toxicity of the nanostructures [24,25]. We addressed this issue via performing a series of biophysical analyses and visualizations, showing that GO water dispersion is a typical colloid.

Firstly, we assessed the size of GO particles using DLS. Our data indicate relative homogeneity of the sample, as evidenced by a PdI value of approximately 0.29 and two narrow peaks representing hydrodynamic diameters of the GO aggregates of approximately 128 nm and 389 nm. The obtained parameters indicate that the sample is homogenous for biomedical applications (PdI value below 0.3), and therefore a good candidate for further research in the field [26]. Additionally, AFM and SEM visualization revealed the presence of GO layers with heights of up to 1 nm in the analyzed sample. The obtained results indicate good stability of the analyzed GO water dispersion in the given experimental conditions and storage period (18 months). These parameters warrant further consideration of water dispersion as a potential candidate for diverse biomedical applications, including drug delivery [27,28].

Subsequently, we assessed the biological effects of GO water dispersion in vitro. Firstly, we employed *Salmonella enterica* serovar Typhimurium TA98 and TA102 strains in the Ames test, to verify mutagenic activity of GO. The results of analyses in both strains revealed no significant difference in the number of revertants between negative controls and any of the GO samples, indicating no mutagenic activity of the analyzed particles. The obtained data are in full agreement with the evaluation of another carbon-based nanostructure, C_60_ fullerene, which was similarly shown to be non-mutagenic, although only in the *S. enterica* TA98 strain [29]. Use of two diverse strains of bacteria during the mutagenicity evaluation allowed us not only to assess GO’s potential to induce frameshift mutations (verified in the TA98 strain) but also transitions/transversions (TA102 strain) [19], providing additional evidence on the biosafety of GO.

The promising results of the mutagenicity test were further confirmed and deepened via the AlamarBlue cytotoxicity test. In this, we observed a reduction of metabolic activity in the cancerous MelJuSo melanoma cell line, indicating cytotoxic activity of GO. These data are especially interesting when juxtaposed with the results of the corresponding experiment conducted using the non-cancerous HaCaT cell line—in this case, metabolic activity of the cells was increased. The mechanism behind this phenomenon remains elusive; however, it may be speculated that, similarly to water-soluble C_60_ fullerene particles, GO induces reactive oxygen species (ROS) generation in the cells. Generated ROS, in consequence, kill cancerous cells, whose metabolic activity is already elevated, while in case of non-cancerous cells, metabolism intensifies to scavenge ROS [30]. These biological data reveal the remarkable potential of GO nanostructures in the field of biomedicine, especially in anticancer therapy, as a drug delivery vessel with the potential to modulate and enhance anticancer drugs’ cytotoxic activity.

Nevertheless, the potential application of GO still requires answers to concerns over its toxicity and adverse effects on living organisms. Regardless of the route of nanoparticles administration, they will interact with blood cells and plasma proteins eventually. Therefore, the assessment of the blood compatibility of GO and any of its derivatives is a matter of the utmost importance.

The most pronounced toxic effect disqualifying novel pharmaceuticals from further research and use in medicine is hemolysis. Therefore, we decided to evaluate the hemolytic potential of GO via the quantification of the hemoglobin released from GO-exposed rabbit erythrocytes. Our results indicate no hemolytic effects of GO in low doses of up to 5 µg/mL. However, higher doses, especially 50 µg/mL and 100 µg/mL, are characterized by a significant degree of erythrocyte hemolysis, reaching 57% for the highest dose, which is consistent with literature data [31,32]. Interestingly, in the case of the GO precursor material—graphite—Liao et al. reported only a marginal, nonsignificant increase of erythrocyte hemolysis, reaching not more than 3%, even in doses as high as 200 µg/mL, attributing this phenomenon to the hydrophobic surface of graphite [31]. These data indicate that GO, unlike graphite, compromises erythrocytes cell membranes, inducing strong, electrostatic interactions between positively charged membrane phospholipids and negatively charged oxygen groups on the GO particle’s surface. Therefore, we can hypothesize that proper and efficient functionalization of the GO surface may be an effective strategy to increase its biocompatibility by decreasing hemotoxicity and, in consequence, facilitate the application of GO in the field of biomedicine.

However, the use of carbon-based nanoparticles, such as GO, in biomedicine also faces other limiting factors, including disrupting platelet functions. Platelet dysfunctions may lead to life-threatening conditions, as platelets, besides their primary, hemostatic function, also play a crucial role in the homeostatic processes [33].

Therefore, in this study, we analyzed GO’s influence on these cells’ functions. Our results indicate that GO in high concentrations (50 µg/mL and 100 µg/mL) decreases both kinetics and intensity of ADP-induced platelet aggregation after 1 h incubation. Moreover, we demonstrated that GO affects platelet disaggregation in a manner dependent on the incubation time and nanostructure concentration. Specifically, after 5 min of incubation, platelet disaggregation is significantly inhibited by high GO concentrations, while after 60 min of incubation, lower concentrations of GO demonstrate similar effects (10, 50, and 100 µg/mL and 1 and 5 µg/mL, respectively). The cause of this phenomenon is elusive; however, we can speculate that in the case of lower GO concentrations, they require longer incubation to exhibit inhibitory effects, while the effects of higher concentrations are shifted towards aggregation processes. Interestingly, the literature data on this effect are inconsistent. Podolska et al. showed that GO in concentrations as high as 50 µg/mL does not influence the aggregation of platelets in rabbit platelet-rich plasma (PRP) [34]. However, Singh et al. demonstrated that GO may induce integrin-mediated platelet aggregation both in vitro and in vivo, to the extent observed previously for thrombin, one of the most potent platelet agonists [35,36]. These inconsistencies may probably be attributed to the diverse size and topography of the GO particles, originating from the differences in the synthesis method.

Furthermore, a recent study suggested the thrombogenic action of GO, which can potentially interact with plasma coagulation factors [37]. We decide to investigate this phenomenon with common methods for blood clotting analysis, namely PT (the prothrombin time test), TT (the thrombin time test), and aPTT (the activated partial thromboplastin time test). Values obtained in these tests describe the time required for clot formation in rabbit plasma samples. Our results indicate that all three parameters, after blood incubation with GO in a wide concentrations range, do not differ significantly from control values. Therefore, we can conclude that GO at the tested concentrations (0.01 to 10 µg/mL) does not trigger the coagulation cascade in rabbit plasma in vitro. This is further confirmed by the literature data. Podolska et al. reported a lack of GO’s influence on aPTT and TT values in human plasma [34], while Feng et al. showed only a very small effect on the PT value; however, the aPTT value increased slightly in plasma with a high GO concentration [38]. On this basis, we can speculate that GO influences the intrinsic but not the extrinsic coagulation pathway, probably as a result of the particles’ interactions with plasma clotting factors.

## 5. Conclusions

Despite the growing interest in the application of GO, both in industry and biomedicine, there is still limited information on the potential toxicity and side effects in humans. The biocompatibility data are missing, although required for the development of effective agents for medicinal use. The existing reports on these structures indicate that their toxicity is closely correlated with the size and physicochemical properties of the particle surface, as they will inevitably contact blood components and body cells. This phenomenon, however challenging, provides scientists with the possibility to overcome potential problems via alterations in synthesis methods and surface functionalization. Nevertheless, a deep understanding of GO’s effects on blood cells and plasma proteins is required for further progress in the application of this nanostructure.

Therefore, in the present study, we analyzed and characterized a GO water dispersion using spectroscopic and microscopic methods. Subsequently, we assessed the mutagenicity and cytotoxicity of the GO using appropriate in vitro assays. Our data indicate a lack of mutagenic GO activity in the Ames test, while cytotoxic activity was only observed in the case of the cancerous melanoma cell line, and metabolism and viability of human keratinocytes were elevated. The latter phenomenon can probably be attributed to GO-induced ROS generation. Furthermore, we investigated the biological effects of GO in blood, including hemolysis, platelet aggregation, and plasma coagulation. Plasma coagulation analysis reveal no influence of GO in a wide concentration range (0.01–10 μg/mL) on PT, TT and aPTT values. However, higher concentrations of GO particles (≥50 μg/mL) can affect the blood cells, including erythrocytes and platelets, changing RBC membrane integrity and influencing the process of platelet aggregation. These findings suggest that GO particles are promising platforms for drug delivery, with the potential to improve cancer therapy in the future, but also for bioimaging and phototherapy, antimicrobial coatings, and tissue engineering.

## Figures and Tables

**Table 1:** In vitro effects of GO on ADP-induced platelet aggregation.

		ADP-Induced Platelet Aggregation Parameters			
		*A_max_* (%)	*V*_0_ (%·s^−1^)	*T_max_* (s)	*A_6min_* (%)
5 min incubation of PRP with GO particles					
control		53 ± 2	1.2 ± 0.2	100 ± 7	30 ± 2
GO concentration (μg/mL)	1	55 ± 2	1.2 ± 0.1	95 ± 5	30 ± 1
	5	54 ± 1	1.1 ± 0.2	110 ± 6	35 ± 2
	10	55 ± 2	1.1 ± 0.1	110 ± 4	45 ± 3 *
	50	56 ± 3	1.1 ± 0.1	118 ± 5	49 ± 2 *
	100	57 ± 3	0.9 ± 0.1	120 ± 8	55 ± 3 *
60 min incubation of PRP with GO particles					
control		55 ± 3	0.9 ± 0.2	95 ± 5	35 ± 2
GO concentration (μg/mL)	1	60 ± 3	1.0 ± 0.1	100 ± 5	55 ± 3 *
	5	60 ± 2	1.1 ± 0.2	95 ± 4	55 ± 2 *
	10	55 ± 2	0.9 ± 0.1	110 ± 6	49 ± 3 *
	50	45± 2 *	0.8 ± 0.1	115 ± 7 *	39 ± 2
	100	43 ± 2 *	0.6 ± 0.1 *	140 ± 5 *	40 ± 3

*A_max_* (%)—maximum aggregation; *V*_0_ (%·s^−1^)—initial aggregation velocity; *T_max_* (s)—time required to reach *A_max_*; *A_6min_* (%)—aggregation level 6 min after ADP supplementation; All results were expressed as means ± standard deviation. Data were normally distributed (Kolmogorov–Smirnov test); * significant difference from control (*p* < 0.05).

**Table 2:** In vitro effects of GO on coagulation potential of rabbit plasma.

		Coagulation Tests		
		aPPT	PT	TT
5 min incubation of rabbit plasma with GO particles				
control		16.9 ± 1.3	7.9 ± 0.9	11.3 ± 1.2
GO concentration (μg/mL)	0.01	18.7 ± 1.3	8.8 ± 0.5	9.3 ± 1.1
	0.05	20.6 ± 0.9	7.4 ± 0.7	8.8 ± 1.3
	0.1	17.4 ± 0.5	7.3 ± 0.9	9.0 ± 0.9
	0.5	16.5 ± 1.1	7.4 ± 0.6	8.9 ± 1.1
	1	16.1 ± 0.9	8.5 ± 0.8	8.5 ± 0.9
	5	15.9 ± 1.3	7.3 ± 0.6	8.3 ± 0.6
	10	17.8 ± 1.2	7.2 ± 0.7	11.0 ± 0.7
30 min incubation of rabbit plasma with GO particles				
control		17.6 ± 1.2	8.8 ± 0.7	7.0 ± 0.7
GO concentration (μg/mL)	0.01	15.5 ± 1.2	8.1 ± 0.9	8.2 ± 0.8
	0.05	15.6 ± 1.3	6.9 ± 1.0	6.8 ± 0.9
	0.1	18.4 ± 1.1	7.7 ± 0.8	7.7 ± 0.5
	0.5	17.8 ± 1.2	7.3 ± 0.7	7.8 ± 0.4
	1	20.4 ± 0.9	7.0 ± 0.9	8.3 ± 0.5
	5	16.0 ± 0.8	7.4 ± 1.1	7.5 ± 0.3
	10	19.1 ± 1.1	6.9 ± 0.8	7.8 ± 0.5

PT—results of prothrombin time test, TT—results of thrombin time test, aPTT—results of activated partial thromboplastin time test.

## Data Availability

The original contributions presented in this study are included in the article. Further inquiries can be directed to the corresponding authors.

## References

[1] Ikram R., Jan B.M., Ahmad W. (2020). An Overview of Industrial Scalable Production of Graphene Oxide and Analytical Approaches for Synthesis and Characterization. J. Mater. Res. Technol..

[2] Shin D.S., Kim H.G., Ahn H.S., Jeong H.Y., Kim Y.J., Odkhuu D., Tsogbadrakh N., Lee H.B.R., Kim B.H. (2017). Distribution of Oxygen Functional Groups of Graphene Oxide Obtained from Low-Temperature Atomic Layer Deposition of Titanium Oxide. RSC Adv..

[3] Jiříčková A., Jankovský O., Sofer Z., Sedmidubský D. (2022). Synthesis and Applications of Graphene Oxide. Materials.

[4] Yu W., Sisi L., Haiyan Y., Jie L. (2020). Progress in the Functional Modification of Graphene/Graphene Oxide: A Review. RSC Adv..

[5] AbouAitah K., Sabbagh F., Kim B.S. (2023). Graphene Oxide Nanostructures as Nanoplatforms for Delivering Natural Therapeutic Agents: Applications in Cancer Treatment, Bacterial Infections, and Bone Regeneration Medicine. Nanomaterials.

[6] Lee J., Kim J., Kim S., Min D.H. (2016). Biosensors Based on Graphene Oxide and Its Biomedical Application. Adv. Drug Deliv. Rev..

[7] Esmaeili Y., Bidram E., Zarrabi A., Amini A., Cheng C. (2020). Graphene Oxide and Its Derivatives as Promising In-Vitro Bio-Imaging Platforms. Sci. Rep..

[8] Seifi T., Reza Kamali A. (2021). Antiviral Performance of Graphene-Based Materials with Emphasis on COVID-19: A Review. Med. Drug Discov..

[9] Shafiee A., Iravani S., Varma R.S. (2022). Graphene and Graphene Oxide with Anticancer Applications: Challenges and Future Perspectives. MedComm.

[10] Shi H., Zhang B., Liu S., Tan C., Tan Y., Jiang Y. (2018). A New Strategy Involving the Use of Peptides and Graphene Oxide for Fluorescence Turn-on Detection of Proteins. Sensors.

[11] Marković Z.M., Jovanović S.P., Mašković P.Z., Mojsin M.M., Stevanović M.J., Danko M., Mičušík M., Jovanović D.J., Kleinová A., Špitalský Z. (2019). Graphene Oxide Size and Structure Pro-Oxidant and Antioxidant Activity and Photoinduced Cytotoxicity Relation on Three Cancer Cell Lines. J. Photochem. Photobiol. B.

[12] Maleki M., Zarezadeh R., Nouri M., Sadigh A.R., Pouremamali F., Asemi Z., Kafil H.S., Alemi F., Yousefi B. (2021). Graphene Oxide: A Promising Material for Regenerative Medicine and Tissue Engineering. Biomol. Concepts.

[13] Sukhodub L., Fediv V., Kumeda M., Sukhodub L., Kulchynskyi V., Tkachuk I., Cherepanov V., Prylutskyy Y. (2023). Electrical Properties of Biodegradable Chitosan-Calcium Phosphate Nerve Conduits Doped with Inorganic Nanoparticles. Colloids Surf. A Physicochem. Eng. Asp..

[14] Jiang T., Amadei C.A., Lin Y., Gou N., Rahman S.M., Lan J., Vecitis C.D., Gu A.Z. (2021). Dependence of Graphene Oxide (Go) Toxicity on Oxidation Level, Elemental Composition, and Size. Int. J. Mol. Sci..

[15] Malaikozhundan B., Vinodhini J., Palanisamy S., Manivannan N. (2023). Toxicity Aspects of Nanomaterials. Handbook of Green and Sustainable Nanotechnology: Fundamentals, Developments and Applications: Volume 1–4.

[16] Kiew S.F., Kiew L.V., Lee H.B., Imae T., Chung L.Y. (2016). Assessing Biocompatibility of Graphene Oxide-Based Nanocarriers: A Review. J. Control. Release.

[17] Palmieri V., Perini G., De Spirito M., Papi M. (2019). Graphene Oxide Touches Blood: In Vivo Interactions of Bio-Coronated 2D Materials. Nanoscale Horiz..

[18] Zhang X., Yin J., Peng C., Hu W., Zhu Z., Li W., Fan C., Huang Q. (2011). Distribution and Biocompatibility Studies of Graphene Oxide in Mice after Intravenous Administration. Carbon.

[19] Mortelmans K., Zeiger E. (2000). The Ames Salmonella/Microsome Mutagenicity Assay. Mutat. Res..

[20] Woziwodzka A., Gwizdek-Wiśniewska A., Piosik J. (2011). Caffeine, Pentoxifylline and Theophylline Form Stacking Complexes with IQ-Type Heterocyclic Aromatic Amines. Bioorg. Chem..

[21] Halenova T., Raksha N., Savchuk O., Ostapchenko L., Prylutskyy Y., Ritter U., Scharff P. (2020). Evaluation of the Biocompatibility of Water-Soluble Pristine C60 Fullerenes in Rabbit. Bionanoscience.

[22] Ochedowski O., Bussmann B.K., Schleberger M. (2014). Graphene on Mica-Intercalated Water Trapped for Life. Sci. Rep..

[23] Nemes-Incze P., Osváth Z., Kamarás K., Biró L.P. (2008). Anomalies in Thickness Measurements of Graphene and Few Layer Graphite Crystals by Tapping Mode Atomic Force Microscopy. Carbon.

[24] Singh D.P., Herrera C.E., Singh B., Singh S., Singh R.K., Kumar R. (2018). Graphene Oxide: An Efficient Material and Recent Approach for Biotechnological and Biomedical Applications. Mater. Sci. Eng. C Mater. Biol. Appl..

[25] Volkov Y., McIntyre J., Prina-Mello A. (2017). Graphene Toxicity as a Double-Edged Sword of Risks and Exploitable Opportunities: A Critical Analysis of the Most Recent Trends and Developments. 2d Mater..

[26] Danaei M., Dehghankhold M., Ataei S., Hasanzadeh Davarani F., Javanmard R., Dokhani A., Khorasani S., Mozafari M.R. (2018). Impact of Particle Size and Polydispersity Index on the Clinical Applications of Lipidic Nanocarrier Systems. Pharmaceutics.

[27] Itoo A.M., Vemula S.L., Gupta M.T., Giram M.V., Kumar S.A., Ghosh B., Biswas S. (2022). Multifunctional Graphene Oxide Nanoparticles for Drug Delivery in Cancer. J. Control. Release.

[28] Matvienko T., Sokolova V., Prylutska S., Harahuts Y., Kutsevol N., Kostjukov V., Evstigneev M., Prylutskyy Y., Epple M., Ritter U. (2018). In Vitro Study of the Anticancer Activity of Various Doxorubicin-Containing Dispersions. Bioimpacts.

[29] Borowik A., Prylutskyy Y., Kawelski Ł., Kyzyma O., Bulavin L., Ivankov O., Cherepanov V., Wyrzykowski D., Kaźmierkiewicz R., Gołuński G. (2018). Does C 60 Fullerene Act as a Transporter of Small Aromatic Molecules?. Colloids Surf. B Biointerfaces.

[30] Skivka L.M., Prylutska S.V., Rudyk M.P., Khranovska N.M., Opeida I.V., Hurmach V.V., Prylutskyy Y.I., Sukhodub L.F., Ritter U. (2018). C60 Fullerene and Its Nanocomplexes with Anticancer Drugs Modulate Circulating Phagocyte Functions and Dramatically Increase ROS Generation in Transformed Monocytes. Cancer Nanotechnol..

[31] Liao K.H., Lin Y.S., MacOsko C.W., Haynes C.L. (2011). Cytotoxicity of Graphene Oxide and Graphene in Human Erythrocytes and Skin Fibroblasts. ACS Appl. Mater. Interfaces.

[32] Wang Y., Zhang B., Zhai G. (2016). The Effect of Incubation Conditions on the Hemolytic Properties of Unmodified Graphene Oxide with Various Concentrations. RSC Adv..

[33] Chaudhary P.K., Kim S., Kim S. (2022). An Insight into Recent Advances on Platelet Function in Health and Disease. Int. J. Mol. Sci..

[34] Podolska M.J., Barras A., Alexiou C., Frey B., Gaipl U., Boukherroub R., Szunerits S., Janko C., Muñoz L.E. (2020). Graphene Oxide Nanosheets for Localized Hyperthermia-Physicochemical Characterization, Biocompatibility, and Induction of Tumor Cell Death. Cells.

[35] Singh S.K., Singh M.K., Kulkarni P.P., Sonkar V.K., Grácio J.J.A., Dash D. (2012). Amine-Modified Graphene: Thrombo-Protective Safer Alternative to Graphene Oxide for Biomedical Applications. ACS Nano.

[36] Singh S.K., Singh M.K., Nayak M.K., Kumari S., Shrivastava S., Grácio J.J.A., Dash D. (2011). Thrombus Inducing Property of Atomically Thin Graphene Oxide Sheets. ACS Nano.

[37] Kenry, Loh K.P., Lim C.T. (2016). Molecular Interactions of Graphene Oxide with Human Blood Plasma Proteins. Nanoscale.

[38] Feng R., Yu Y., Shen C., Jiao Y., Zhou C. (2015). Impact of Graphene Oxide on the Structure and Function of Important Multiple Blood Components by a Dose-Dependent Pattern. J. Biomed. Mater. Res. A.

